# Extensive epigenetic and transcriptomic variability between genetically identical human B-lymphoblastoid cells with implications in pharmacogenomics research

**DOI:** 10.1038/s41598-019-40897-9

**Published:** 2019-03-20

**Authors:** Lilla Ozgyin, Attila Horvath, Zsuzsanna Hevessy, Balint L. Balint

**Affiliations:** 1 Genomic Medicine and Bioinformatic Core Facility, Department of Biochemistry and Molecular Biology, Faculty of Medicine, University of Debrecen, H-4032 Debrecen, Hungary; 2 Department of Laboratory Medicine, Faculty of Medicine, University of Debrecen, H-4032 Debrecen, Hungary

## Abstract

Genotyped human B-lymphoblastoid cell lines (LCLs) are widely used models in mapping quantitative trait loci for chromatin features, gene expression, and drug response. The extent of genotype-independent functional genomic variability of the LCL model, although largely overlooked, may inform association study design. In this study, we use flow cytometry, chromatin immunoprecipitation sequencing and mRNA sequencing to study surface marker patterns, quantify genome-wide chromatin changes (H3K27ac) and transcriptome variability, respectively, among five isogenic LCLs derived from the same individual. Most of the studied LCLs were non-monoclonal and had mature B cell phenotypes. Strikingly, nearly one-fourth of active gene regulatory regions showed significantly variable H3K27ac levels, especially enhancers, among which several were classified as clustered enhancers. Large, contiguous genomic regions showed signs of coordinated activity change. Regulatory differences were mirrored by mRNA expression changes, preferentially affecting hundreds of genes involved in specialized cellular processes including immune and drug response pathways. Differential expression of *DPYD*, an enzyme involved in 5-fluorouracil (5-FU) catabolism, was associated with variable LCL growth inhibition mediated by 5-FU. The extent of genotype-independent functional genomic variability might highlight the need to revisit study design strategies for LCLs in pharmacogenomics.

## Introduction

In the past few years, the biomedical research community empowered by high-throughput technologies have gained a broader appreciation for the importance of intra- and inter-cell line variability and dynamics. Genetic diversification may be a driving force behind single-cell heterogeneity and the evolution of genetically unstable cancer cell lines, affecting gene regulatory pathways that play a role in the response to various external cues, including drug response^1^. At the same time, past exposure and cell line handling, as well as intrinsic gene regulatory network state variability of single cells and local differences in cell culture conditions combine to cell population-level phenotypic readouts in bulk experiments^2–6^. A recent study by Hastreiter *et al*. has demonstrated the temporal dynamics of embryonic stem cell (ESC) line heterogeneity due to a switch from one culturing method to another. Exposing ESCs maintained in serum and leukemia inhibitory factor (LIF) to a dual inhibition cocktail (2i; GSK3 and MEK inhibitors) stabilises Nanog expression across single cells of the ES cell culture by inducing Nanog expression and selecting against Nanog-low cells^2^. A thorough understanding of intra- and inter-cell line variability of widely used cell lines and their potential experimental relevance should enable rational experimental design and help draw appropriate conclusions.

Various studies with diverse research aims have taken advantage of the human B-lymphoblastoid cell line (LCL) model over the past few decades. To name a few, LCLs have been used to study the molecular mechanisms of and immunological responses to EBV infection^7–10^, carcinogen sensitivity and DNA repair^11,12^, chromatin organisation and gene expression regulation^13–17^, as well as drug response^18^, mostly in association with the genetic background of the cells. Moreover, LCLs have also been found instrumental in studying non-EBV-related diseases, such as amyotrophic lateral sclerosis^19^, diabetic retinopathy^20^, and bipolar disorder^21^. The main advantages of this cell line model include easy culturing and manipulation, stable karyotype, and publicly available genotype information for a large number of LCLs for molecular association and pharmacogenomics. Given the extensive use of LCLs, it is of fundamental importance to unveil their limitations in order to drive well-informed decisions regarding experimental design.

The LCL model is relatively well-characterised due to studies aimed at uncovering its limitations as B cell surrogates. Several studies have pointed out that LCLs harbour negligible genotypic alterations in continuous culture^22–25^, due in part to the episomal location of the viral genome and limited expression of viral genes^26–28^. As LCLs are derived from primary B cells by means of *in vitro* transformation with the Epstein-Barr virus (EBV), virus-triggered perturbations of molecular pathways and adaptation to culturing conditions are expected. The number of EBV genomes do not substantially vary across cell lines, with more than 90 percent of LCLs carrying 20 to 27 copies^29^, although the EBV copy number has not been shown to alter viral protein expression levels^30^, and only a subtle correlation has been found between viral protein expression and the host cell’s gene expression patterns^29^. The activation of the NF-κB pathway has been revealed to play a major role in transforming the resting B cells^31–33^. Also, Notch signaling-related RBPJκ has been shown to interact with the viral EBNA2, ultimately leading to growth de-repression^34^. Although gene expression profiles clearly discriminate transformed and non-transformed B cell populations, it is worth noting that inter-individual gene expression variation is maintained after EBV transformation^35,36^. DNA methylation studies have shown that differentially methylated regions between LCLs and genotype-matched primary white blood cells are more likely to be hypomethylated in LCLs, but the effects of neither short-term culturing nor freeze-thawing are profound^36–38^. Based on the above observations, many studies assume that LCLs from different individuals offer a good model for how genotypes affect functional genomic patterns and the response to environmental cues, including various pharmaceuticals.

Genotyped LCLs have been instrumental in genomic association studies aimed at finding candidate genomic regions affecting molecular and drug response phenotypes. Using LCLs, quantitative trait loci (QTLs) have been identified, for instance, for transcription factor (TF) binding (tfQTLs), histone modifications (hmQTLs), DNA methylation (mQTLs) and gene expression (eQTLs)^14,16,17,39–41^, proving the model’s feasibility in molecular association studies. Also, LCLs provide an appealing means for pharmacogenomics as they are free of *in vivo* confounders such as age and polypharmacy, and drugs with a narrow therapeutic index can also be tested. Also, at least half of the catalogued genes are expressed in LCLs, rendering it an appropriate model for finding associations between genetic and transcriptomic signatures versus drug response for lymphoproliferative and non-B cell-related diseases^42^. In order to reduce the number of false positives in pharmacogenomic studies, a triangle model has been proposed^43^. Assuming genotype-dependent RNA expression level changes upstream of drug sensitivity variability, the triangle approach includes finding significant genotype-drug response associations, subsequently validated using genotype-RNA expression (eQTL), and gene expression-drug response associations. However, excluding common confounders such as cell culture age and culturing condition differences, there still remains the potential confounding effect of cell line variability of non-genetic origin.

The extent to which non-genetic factors influence chromatin level modifications, gene expression, and ultimately response to environmental signals in LCLs has not yet been evaluated. However, the preparation of distinct LCL batches from the same individual provides an exceptional opportunity to highlight the extent and nature of functional genomic variability independent of the genomic context. In our study, we used a model of five LCLs derived from the same individual (isogenic, hence bearing identical genomic material), prepared in the same laboratory using the same EBV strains to characterise inter-cell line variability at the epigenetic, transcriptomic, and protein surface marker levels. Our results have implications for pharmacogenomics research, supporting a more widespread adoption of the triangle model, which considers baseline RNA levels in LCL-based study design in pharmacogenomics.

## Results

### Basic cell line characterisation

**Figure 1:**
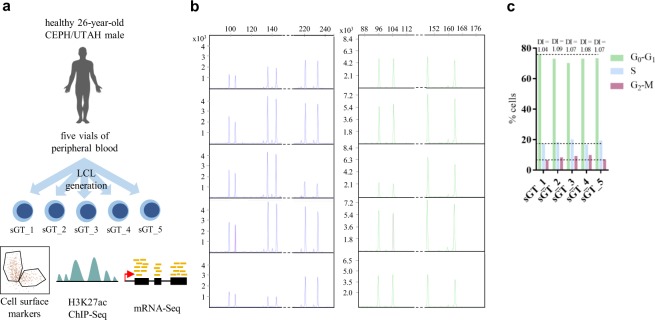
Study design and basic characteristics of sGT LCLs. (**a**) Study design. (**b**) Capillary electropherograms of PCR-amplified STR regions. (**c**) The fraction of cells at each cell cycle stage based on DNA content and calculated DNA-indices (DIs).

In this study, five isogenic LCLs (GM22647-GM22651, denoted as sGT_1 through sGT_5, respectively) derived from the same male individual with European ancestry (CEPH/UTAH)^44^ were used as a model for assessing the extent and nature of isogenic LCL variability driven by non-genetic factors at multiple phenotype levels (Fig. 1a). These cell lines were prepared in the same laboratory, using the same EBV strain, from different collection tubes of blood and were shown to be highly concordant at the level of SNVs and indels and no extensive mosaicism or chromosomal aberration was observed^44^. All cell lines were handled together from preparation for shipment at Coriell Institute, through all culturing, biobanking and experimental steps, that is, they were exposed to the same environment throughout the study (see Materials and Methods). Using cell lines prepared from the same individual and excluding cell culturing-related confounders, sGT LCLs represent a suitable model for exploring molecular phenotype variability among LCLs.

Prior to assessing molecular variability, we wanted to ensure that sGT LCLs possess the same genetic background, are free of mycoplasma infection, and show similar cell cycle progression. The same genetic background and the presence of both types of sex chromosomes were confirmed by amplifying four autosomal polymorphic short tandem repeat (STR) regions and the allosomal Amelogenin locus (AMELX and AMELY), respectively (Fig. 1b); the numerical data are given in Supplementary Table 1. Mycoplasma contamination of cell cultures is a widespread phenomenon which seriously alters cellular behavior and thereby easily invalidates research data. Working batches of sGT LCLs were confirmed to be mycoplasma-free using a PCR-based method (see Materials and Methods section) (data not shown). The fraction of cells in each cell cycle stage (G_0_-G_1_, S, and G_2_-M) was similar for all cell populations, and all calculated DNA-indices (DNA content per nucleus per haploid genome size) were below 1.1, indicating euploidy (Fig. 1c). These results suggest that the selected cell lines are suitable for the purposes of the present research.

**Table 1:** Protein-level expression of selected leukocyte antigens in sGT LCLs as assessed by flow cytometry.

Marker	sGT_1	sGT_2	sGT_3	sGT_4	sGT_5
CD19	+	+	+	+	+
CD20	+	+	+	+	+
CD22	+	+	+	+	+
CD23	+	+	+	+	+
CD45	+	+	+	+	+
HLADR	+	+	+	+	+
CD21	dim	dim	dim	dim	dim
CD43	dim	dim	dim	dim	dim
FMC7	dim	dim	dim	dim	dim
CD10	−	−	−	−	−
CD34	−	−	−	−	−
CD5	−	−	−	−	−
CD79b	−	−	−	−	−
nTdT	−	−	−	−	−
CD24	0.6%	1.1%	1.2%	1.9%	1%
CyIgM	1%	84%	36%	15%	10%
Lambda	5%	59%	36%	dim	13%
Kappa	95%	36%	60%	−	86%
CD81	54%	54%	58%	74%	76%
CD38	87%	87%	63%	20%	64%
cell type	**B cell**	**B cell**	**B cell**	**B cell**	**B cell**

### Probing protein surface markers of sGT LCLs by flow cytometry reveals mature B cell immunophenotype and intra-cell-line heterogeneity

**Figure 2:**
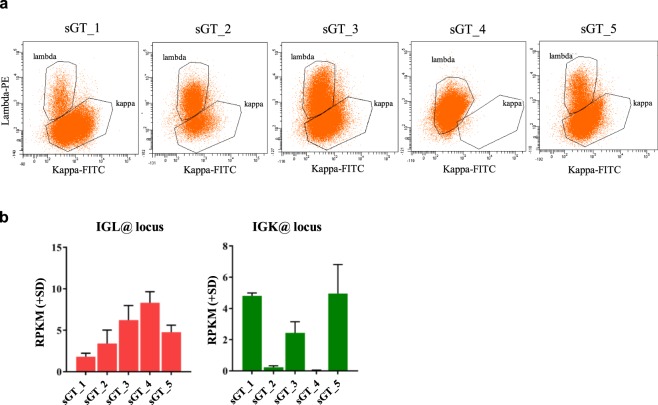
sGT LCL clonality assessed based on kappa-lambda light chain exclusion. (**a**) The dot plots display the fluorescence intensity of individual cells stained with Lambda-PE and Kappa-FITC, as well as the fraction of events falling into lambda and kappa gates (defining lambda positive and kappa positive populations) for each sGT LCL. (**b**) Expression of the immunoglobulin lambda (IGL@) and immunoglobulin kappa (IGK@) loci at RNA level, based on mRNA-Seq data (N = 2).

As the next step, we characterised sGT LCLs with respect to the expression of selected immune-cell-specific protein markers. Each mature human B cell expresses a single class of immunoglobulin light chain, kappa (κ) or lambda (λ), as a result of allelic exclusion through DNA rearrangement during cell maturation. Light chain restriction is regarded as an indicator of monoclonality in B cell malignancies and is commonly used in clinical practice to characterise such lymphoproliferative disorders. Flow-cytometric analysis of sGT LCLs double-stained with fluorescently labeled anti-kappa and anti-lambda antibodies revealed that four out of the five cell lines had not been derived from a single-cell clone (mixed populations). However, the sGT_4 cells were shown to exclusively expose the lambda chain, at a moderate level (evaluated as dim expression), which is suggestive of either pauciclonality or monoclonality (Fig. 2a). The observed surface expression patterns of the kappa and lambda chains were also supported at the RNA level, by assessing normalised RNA-Seq reads over the *IGL* and *IGK* loci (Fig. 2b).

Immunophenotyping has the ability to reveal population-level characteristics of cell lines at the surface marker expression level. Surface marker expression patterns were found to be typical of mature human B cells (CD19+ with low side scatter, CD20+, CD22+, CD23+, CD45+, HLADR+, dim FMC7+, dim CD21+, dim CD43+, CD5−, CD10−, CD34−, and nTdT−). The pan B cell marker CD24 is virtually absent in all cell lines (0.6–1.9% CD24+ cells), which is consistent with the findings of a previous study reporting the loss of CD24 upon EBV infection^45^. Strikingly, CD79b, encoding the Ig beta component of the B cell Receptor (BCR), is also absent from all cell lines. Cell populations contain 54–76% CD81+ cells, and the activation marker CD38 shows a marked expression difference between the cell lines (20–87%). Additionally, the cell lines were stained positive for cyIgM, marker of the pre-B cell stage, to highly varying degrees, with the percentage of positive cells ranging from 1% (sGT_1) to 84% (sGT_2) (Table 1). These findings indicate that the cells in the sGT LCL populations were derived from human B cells, but for certain markers (CD81, CD38, cyIgM) the percentage of cells in each LCL population varies.

### Marked differences in gene regulatory element activity among sGT LCLs

**Figure 3:**
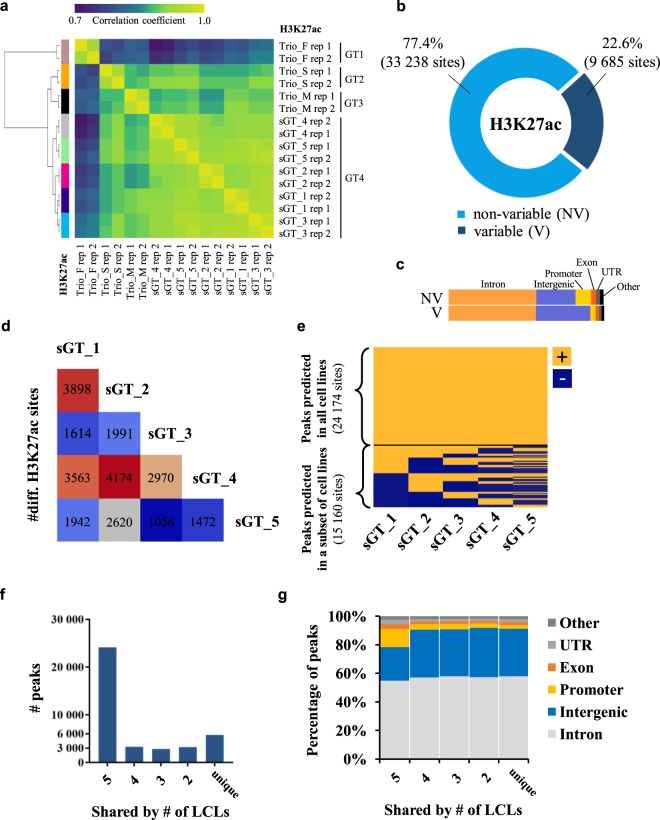
Differential activity of genomic regulatory elements in LCLs as assessed by H3K27ac ChIP-Seq. (**a**) Correlation heatmap based on H3K27ac signals at a consensus set of genomic regions in LCLs derived from four individuals (GT1-GT4): a core CEU trio (GT1-GT3) consisting of a mother (Trio_M), a father (Trio_F) and a male child (son; Trio_S) and GT4, and an unrelated CEU male individual from whom five cell lines were prepared (sGT_1-sGT_5). (**b**) The fraction of the consensus region set showing variable H3K27ac enrichment across the five sGT LCLs (ANOVA, P < 0.05, fold-change >2). (**c**) The fraction of non-variable (NV) and variable (V) H3K27ac peaks annotated to certain genomic region category. (**d**) Pairwise comparison of sGT LCLs, indicating the number of differentially enriched sites per sGT LCL pair. (**e**) Fraction of peaks shared by all or predicted only in a subset of sGT LCLs. (**f**) Number of peaks predicted in all (5), four, three, two, and in only one sGT LCL(s). (**g**) The change in the proportion of certain genomic region categories annotated to peaks with different levels of sharedness.

We profiled the five sGT LCLs with H3K27ac ChIP-Seq to map active regulatory regions over the genome, in biological duplicates (sample-wise ChIP-Seq statistics are provided in Supplementary Table 2). For each cell line, two different vials of the same cell batch were re-cultured and harvested approx. one week apart from each other in order to exclude most of the stochastic changes and capture reproducible differences. A consensus active regulatory region set was derived by merging H3K27ac-enriched regions across the cell lines (excluding singletons), resulting in a set of 42,923 regulatory elements, over which we calculated RPKM values (reads per kilobase per million mapped reads) for each dataset. The RPKM measure enables the within-sample and between-sample comparison of read densities over pre-defined genomic regions by normalising for region length as well as the total number of sequencing reads. Although we found remarkably high adjusted pairwise correlation coefficients across the whole sGT dataset (0.9–0.97), biological replicates clustered together, indicating that these cell lines, despite their genetic homogeneity, harbour unique H3K27ac signatures. When we included LCLs from a CEPH/UTAH trio genetically unrelated to the sGT cells in the analysis, the genetically identical cell lines were clearly separated from trio LCLs (Fig. 3a). We then categorized the consensus set to regions with “non-variable” or “variable” H3K27ac enrichment, and based on our inclusion criteria (RPKM fold-change >2, P < 0.05 in at least one comparison), 9,685 sites (22.6% of all consensus) fell into the variable category (Fig. 3b). Also, as expected, we found a higher within-cell-line than between-cell-line correlation using the r^2^ statistic (the fraction of total variance explained by the linear regression model) (Supplementary Fig. 1). Of note, despite its monoclonality, the sGT_4 cell line did not show a higher within-cell-line correlation compared to the non-monoclonal sGT LCLs. This may indicate that the limited time (approx. one week) sGT LCL replicates - recovered from different vials of the same freezing batch - spent in separate cultures did not allow for a more substantial chromatin-level diversification of non-monoclonal cells than the monoclonal line (sGT_4). When comparing the genomic distribution of variable versus non-variable regions, we found that variable sites predominantly map to intergenic regions (enhancers), while genic regions, especially promoters, are less affected, indicating a regulatory constraint on activity changes at gene-proximal elements (Fig. 3c). Pairwise comparisons revealed that the number of affected regulatory regions range from 1,056 to 4,174 (mean = 2,530; median = 2,305.5) per sGT pair (Fig. 3d). Comparing cell-line-specific consensus regions (by merging those predicted in both replicates), 38.5% (15,160) were found to be shared by 1–4 cell lines, and 5,785 regions were unique to one cell line (Fig. 3e,f). With a decreasing level of sharedness (i.e., with a decreasing number of LCLs containing the given peak), the fraction of promoters also decreases (Fig. 3g). Overall, reproducible differences can be detected at one-fourth of the active regulatory regions in five sGT LCLs, and most of these changes affect distal enhancers.

### Coordinated activity change of regulatory elements over large genomic regions, including clustered enhancers

**Figure 4:**
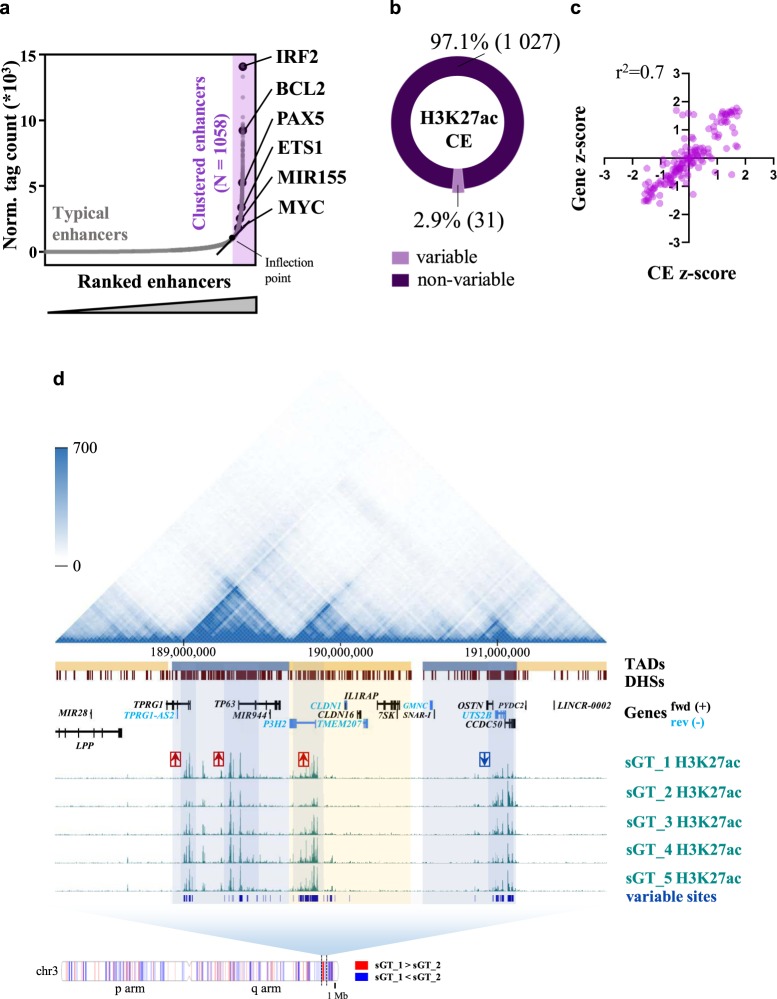
H3K27 acetylation and gene expression variability at the level of clustered enhancers (CEs) and over large genomic regions. (**a**) Normalized H3K27ac ChIP-Seq tag counts over typical and clustered enhancers, ranked by increasing signal; some CE examples are highlighted based on their association with genes related to B-cell-specific and immune functions, or induced by Epstein-Barr virus infection. (**b**) The fraction of CEs showing variable H3K27ac enrichment across the five sGT LCLs (ANOVA, P < 0.05, fold-change >2). (**c**) The scatter plot shows the effect of H3K27ac variability over CEs on the expression of the closest expressed gene; z-scores calculated for CEs and their closest genes were plotted against each other, for all possible comparisons (N = 155). (**d**) The panel shows contact frequencies (HiC), predicted topologically associated domains (TADs) and DNase hypersensitive sites (DHS), as well as genes over a ~4 Mb-long selected region on chromosome 3 in the GM12878 cell line, together with H3K27ac bedGraphs and differentially enriched regions from all five sGT LCLs (blue vertical lines). The distribution of regions differentially enriched between sGT_1 and sGT_2 over chromosome 3, colored based on the direction of change, is shown on the bottom of the panel. Red arrows on the bedGraphs indicate the direction of change between sGT_1 and sGT_2.

In the next stage, we asked whether the activity of super-enhancers (hereon denoted as clustered enhancers, CEs) was also prone to genotype-independent variability or, in contrast to single regulatory elements, their activity remained largely stable across LCLs. CEs are a distinct class of regulatory regions which occupy large – from a few and up to hundreds of kilobases long - genomic regions consisting of enhancers <12.5 Kb apart from each other, and characterised by especially high regulatory activity. As CEs largely control highly expressed genes critical for development, not surprisingly, the loss of certain CEs have already been associated with various diseases^46^. We first predicted consensus LCL CEs using pooled H3K27ac ChIP tags, resulting in a total of 1,058 putative CE regions. In line with the literature, predicted LCL CEs lie in the close proximity of genes involved in B cell-specific functions and immune response, such as the TFs *PAX5* and *IRF2*, as well as genes previously identified as EBV super-enhancers^33^, such as *BCL2*, *ETS1*, *MIR155* and *MYC*, a fraction of which ensure continuous cell proliferation (Fig. 4a). Among the predicted CEs, 518 (49%) contain one or more enhancer elements previously classified as variable, but when taking into account H3K27ac levels over the whole CE region, only 31 (2.9%) were found to be variable (P < 0.05, fold-change >2) across the five cell lines (Fig. 4b), indicating that the activity of individual enhancer elements of CEs may vary without seriously affecting the CE activity per se. Upon inferring the effect of CE activity on the expression of the closest protein-coding gene, we found a linear relationship between the fold-difference of CEs and the fold-difference of the closest gene (Fig. 4c). On Fig. 4d, we show a representative region on chromosome 3 over which multiple gene regulatory elements show variable H3K27ac signal. Interestingly, a switch in signal direction could be observed at one of the visualised TAD (topologically associated domain) boundaries. In line with that, by comparing the RPKM values in sGT_1 and sGT_2 over the whole chromosome, we could identify large regions characterised by coordinated H3K27ac signal change. Notably, despite this seemingly high level of coordinatedness, most of these regions did not overlap with, and were not eligible for, the definition of a CE (for details of CE prediction, please refer to the Materials and Methods section). Inferring the common features of genes associated with variable CEs, among the most enriched biological processes were immune-related functions including leukocyte activation (5.5 * 10^−4^) and leukocyte cell-cell adhesion (5.6 * 10^−4^), while among the most enriched molecular functions were transcription factor activity (P = 1.2 * 10^−2^) and LPS binding (P = 3.2 * 10^−2^). Hence, in contrast to typical enhancers, the overall activity of clustered enhancers show limited variability across sGT LCLs, and the variability at the single CE component-level rarely leads to a significant perturbation of CE activity.

### Differences in H3K27ac levels are linked to transcriptomic changes affecting specialised cellular pathways

**Figure 5:**
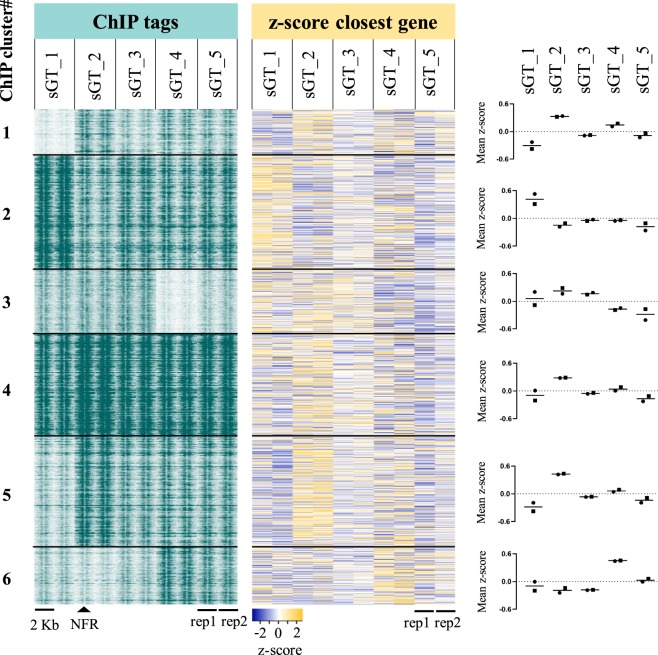
Read distribution heatmap of k-means clustered variable H3K27ac sites (9 685) and z-scores of genes closest to the given H3K27ac region (heatmap). Z-scores were also calculated for the two replicates independently; dots represent mean z-scores for each replicate in each cluster (dot: rep1, square: rep2), while black vertical lines represent means of the two replicates in the corresponding cluster. NFR = nucleosome-free region.

**Figure 6:**
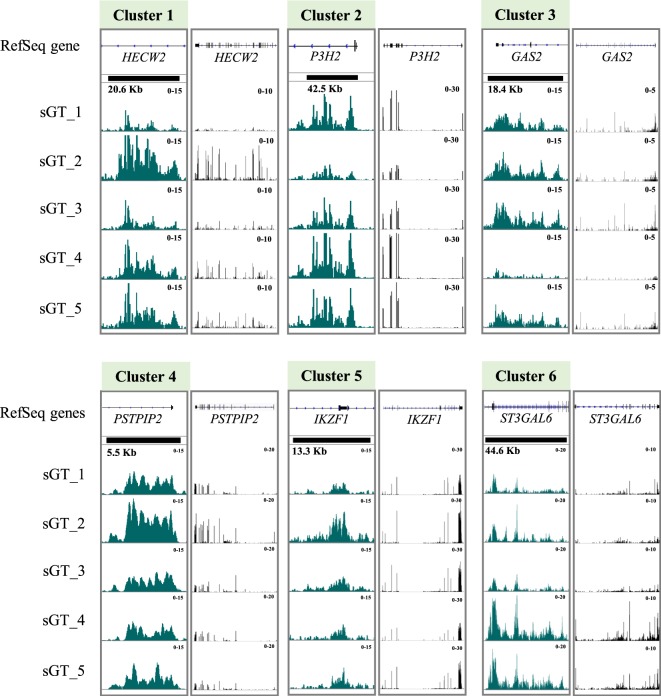
Integrative Genomic Viewer (IGV) snapshot of pooled BedGraph tracks at genomic regions representative of each of the six ChIP clusters (variable H3K27ac regions), and the genes closest to each variable ChIP region. The black rectangles over ChIP-Seq tracks represent the dimensions of each variable region.

**Figure 7:**
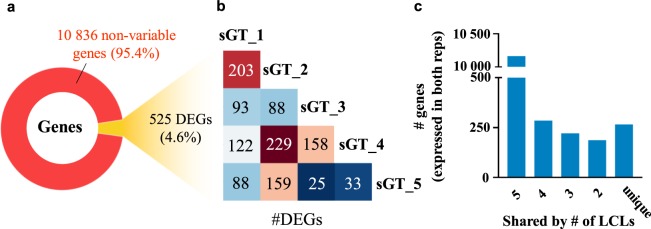
Gene expression variability across sGT LCLs as assessed by mRNA-Seq. (**a**) The fraction of genes showing variable expression levels across the five sGT LCLs (ANOVA, FDR = 0.05, fold-change >2). (**b**) Pairwise comparison of sGT LCLs, indicating the number of differentially expressed genes (DEGs) per sGT LCL pair. (**c**) The number of genes expressed in a certain number of sGT LCLs (CPM >5 in both replicates).

**Figure 8:**
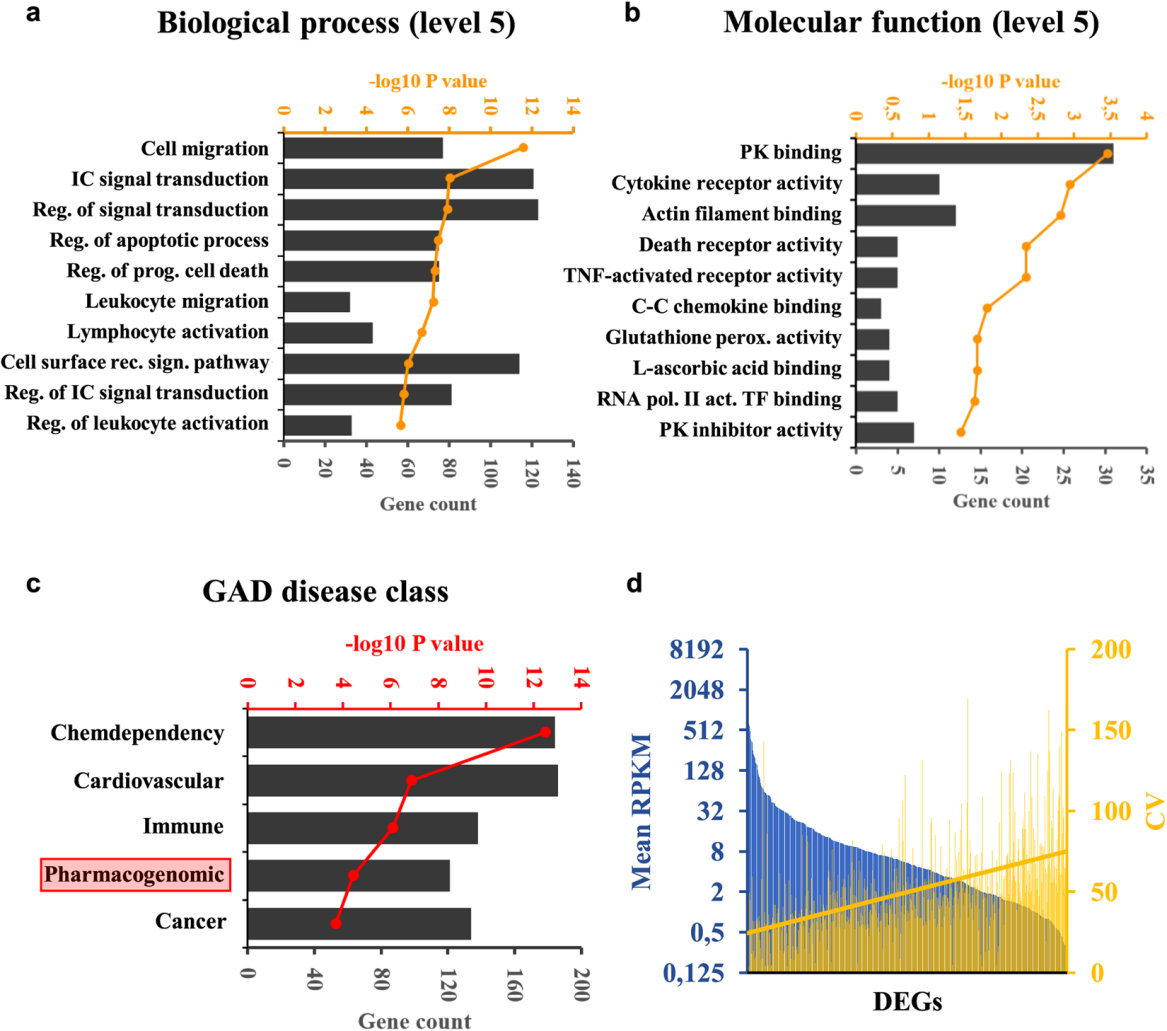
Coefficient of Variance (CV) and Gene Ontology (GO) annotation of DEGs. (**a**) –log 10 P values and gene numbers of the most highly enriched biological processes, (**b**) molecular functions, and (**c**) GAD disease classes among DEGs. (**d**) Mean RPKM of DEGs across all sGT LCLs and their corresponding CV values. The yellow line is the linear fit of CV values.

In order to get a comprehensive overview of the correlation between regulatory activity changes and transcriptomic variability, we performed mRNA-Seq in all five cell lines in biological duplicates. For pairwise comparisons of replicates and cell lines, please refer to Supplementary Fig. 2. First we plotted nucleosome-free region-centered H3K27ac tag counts at the 9,685 variable H3K27ac-enriched genomic elements together with the z-scores of genes closest to each variable site. Prior to plotting, k-means clustering of the variable sites was performed in order to capture the main patterns of regulatory variability, which resulted in 6 definitive clusters. In order to capture the main gene expression patterns across cell lines, we calculated z-scores genewise by subtracting mean RPKM of the given gene from the RPKM of the respective gene of each cell line, and divided this value by the standard deviation. We found that regulatory activity changes are in general followed by gene expression changes (Fig. 5). Figure 6 shows representative examples of each of the 6 clusters, visualising ChIP-Seq and mRNA-Seq bedGraph tracks for selected regulatory regions and their closest genes, respectively, across the five sGT cell lines (Fig. 6). Despite this good overall correlation between ChIP-Seq and mRNA-Seq signals, only 4.6% of genes (525) showed variable expression (denoted as differentially expressed genes; DEGs) across the five cell lines (FDR = 0.05, FC > 2) (Fig. 7a), with 25–229 genes being significant per LCL pair (mean = 119.8; median = 107.5). Considering only those genes that are expressed in both replicates of at least one cell line (CPM > 5), 959 were present in only a subset of cell lines (Fig. 7c). Importantly, none of the genes whose expression has previously been associated with the EBV copy number (*CXCL16*, *AGL*, *ADARB2*) show variable expression in our dataset^29^, suggesting that on the EBV infection level, there is no major difference between these cell lines. Upon performing Gene Ontology (GO) analysis on the DEG set, among the most enriched biological processes were cell migration (P = 2.8 * 10^−12^), intracellular signal transduction (P = 9.8 * 10^−9^), and regulation of apoptotic process (P = 3.4 * 10^−8^), while the most enriched molecular functions include immune receptors and transcription factors (Fig. 8a,b). Surprisingly, upon characterising the affected genes, we found that 121 of the DEGs were categorised as possible pharmacogenes in the Genetic Association Database (GAD) (Fig. 8c). Coefficient of variance (CV) values of genes are inversely proportional to the mean expression level (Fig. 8d); the genes with the highest level of variance encode genes located at the cell surface, have receptor function and play a role in cell motility and signal transduction, while genes with the lowest variance tend to localize inside the cell and mediate immune and apoptotic functions as components of signal transduction pathways (Supplementary Fig. 3). In summary, the extent of RNA-level variability among sGT LCLs is far smaller compared to regulatory element-level variability, which is expected in light of previous studies uncovering widespread enhancer redundancy^47^, and they affect genes that are of considerable interest for those engaged in pharmacogenomics research.

### Non-genetic gene expression changes might affect LCL drug response phenotypes

**Figure 9:**
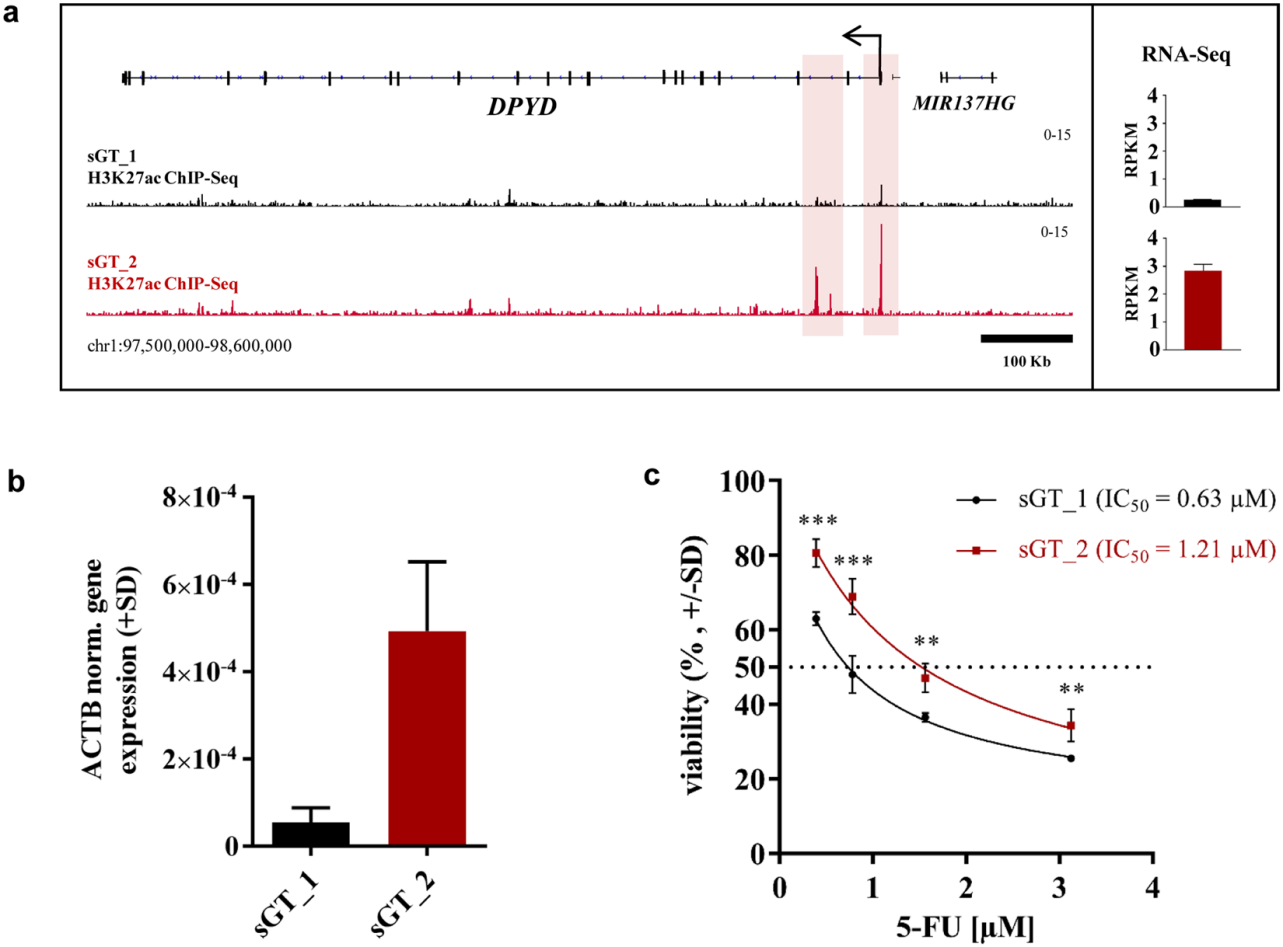
Response of sGT_1 and sGT_2 cell lines to 5-FU. (**a**) IGV snapshot of the genomic region sorrounding the *DPYD* gene (~1 Mb), showing H3K27ac BedGraph tracks pooled from the two replicates for sGT_1 and sGT_2 (with the two tracks on the same scale). DPYD mRNA expression values (RPKM (+SD), N = 2) are also indicated. (**b**) Validation of differential DPYD gene expression between sGT_1 and sGT_2 with RT-qPCR (N = 2, normalized to *ACTB*). (**c**) Dose-response curves representing the decrease of cell viability upon 72-hour treatment with increasing concentrations of 5-FU for sGT_1 and sGT_2 cell lines, using the MTT assay. The half-maximal inhibitory concentration (IC_50_) was calculated using the non-linear regression curve fit using the least-squares method (R^2^_sGT_1_ = 0.97, R^2^_sGT_2_ = 0.95). ***P < 0.001, **P < 0.01.

Uncovering the level of gene expression variability of sGT LCLs, the question arises whether it has relevance for biomedical research. Dihydropyrimidine dehydrogenase (*DPYD*), which catalyses the initial and rate-limiting step of pyrimidine catabolism, has been shown to be involved in the degradation of fluoropyrimidine chemotherapeutic agents, including 5-fluorouracil (5-FU) and its prodrugs, Capecitabine and Tegafur. Inherited *DPYD* deficiency has been linked to severe 5-FU toxicity^48^. Upon inspecting the genomic environment of the *DPYD* gene, three H3K27ac-marked regulatory elements show differential H3K27ac enrichment, which is linked to a significantly different *DPYD* expression between the sGT_1 and sGT_2 cell lines at the RNA level based on mRNA-Seq (sGT_2 > sGT_1, fold-change = 8.5) (Fig. 9a). The differential expression could also be validated by RT-qPCR from total RNA samples isolated from cells harvested independently from those used for RNA-Seq (Fig. 9b). To test whether *DPYD* expression might lead to 5-FU response difference in these cell lines, we treated sGT_1 and sGT_2 cells with different concentrations of 5-FU and assessed cell viability as a measure of cytotoxicity. We found that at each concentration the viability was significantly higher in sGT_2, leading to an almost twofold increase in the IC_50_ value (IC_50 sGT_1_ = 0.63 μM, IC_50 sGT_2_ = 1.21 μM) (Fig. 9c). These results suggest that genotype-independent gene expression variability among LCLs contributes to the cellular response to drugs and may be present as a confounder in LCL-based pharmacogenomic screenings.

## Discussion

The scientific community has made considerable efforts to thoroughly characterise commonly used cell-line models, thus providing invaluable insights into their advantages and limitations, which serves as a foundation for rational experimental design. In parallel with the emergence and maturation of high-throughput sequencing technologies, EBV-transformed human B-lymphoblastoid cell lines have become one of the primary models for identifying QTLs affecting chromatin and transcriptomic features. Moreover, their widely acknowledged advantages have rendered LCLs a reasonable choice for pharmacogenomic studies. Isogenic LCLs prepared from the same healthy individual (sGT LCLs) provide a useful model for uncovering the extent and nature of LCL variability unexplainable by differences in the genetic background or culturing conditions. Our results suggest that LCLs retain an immunophenotype characteristic to B cells and harbour substantial genotype-independent epigenetic and gene expression variability despite strict and consistent cell culturing practice and high genetic stability. Hence, the indiscriminate use of LCLs by being unaware of the extent of molecular phenotype variability unrelated to the genetic makeup of the cells might confound finding relevant genetic associations. Molecular signatures represented by reproducible H3K27ac enrichment and gene expression differences clearly discriminate the cell lines. Despite the good correlation between H3K27ac signatures and the expression of nearby genes, the extensive variability of H3K27ac-marked regions, including components of clustered enhancers, are coupled to a relatively modest change in the level of poly(A)+ RNAs. Among the variably expressed genes are numerous disease-associated and pharmacogenes, as well as components of cellular signaling, genes involved in immune response and lymphocyte-specific activities. At the same time, we showed that selected sGT LCLs with different *DPYD* mRNA levels show 5-FU sensitivity, providing an additional level of evidence that the current and widely adopted model of pharmacogenomic study design needs to be revisited. Our results also suggest that low-passage sGT LCLs represent a mixture of cells derived from multiple successfully transformed parental B cells. Immunophenotyping shed light on the presence of LCL sub-populations with a markedly different surface expression of several probed antigens.

Limitations of continuous cell line models, such as genomic instability and phenotypic drift, have long been acknowledged. However, studies using non-cancerous cell lines are generally based on the assumption that measured cell line characteristics, including genetic makeup, remain largely comparable over time and across laboratories; hence, genetic differences between source individuals might dominate both baseline and triggered molecular phenotype differences. LCLs derived from non-cancerous resting B cells are expected to be genetically and karyotypically more stable than cancer cell lines, as they are less prone to accelerated genetic evolution due to, for instance, deregulated repair mechanisms. Indeed, studies have found that the genomes of most LCLs, including those used in this study, faithfully reflect those of the parental cells at low passage numbers (<20)^22–25,44,49,50^. It should be noted that the cumulative number of passages (N = 14) performed on sGT LCLs used in this study, including subculturing and seed stock preparations, was well below this empirical cutoff. Several studies have been conducted to uncover the level of epigenomic and transcriptomic perturbations in LCLs owing to factors like EBV transformation, culturing, and freeze-thawing. DNA methylation profiles were shown to change between LCLs and parental surrogates, presenting predominantly as hypomethylation at random loci^36,37,51,52^. Of note, probably due to experimental considerations, LCLs were mostly compared to matched peripheral blood mononuclear cells (PBMCs) or peripheral blood lymphocytes (PBLs) as a surrogate for parental B cells. EBV transformation leads to the perturbation of molecular pathways to an extent that based on principal component analysis of gene expression profiles LCLs become more similar to each other than to their parental B cells. However, gene-level expression differences between B cells of different individuals are retained in their derived LCLs^35–37^, which is probably due to a marked change of a relatively limited set of genes driven by EBV proteins with transcription factor activity^33,53^.

Numerous studies used LCLs to uncover the main features and genetic background of inter-individual differences at the chromatin level. These studies identified specific features of transcription factor binding- and histone modification-level variability, such as coordinated changes related to the 3D organisation of the genome and evolutionary implications, in relation to genotypic differences^16,17,54,55^. However, due to study design constraints, genotype-independent differences could not be discriminated from genotype-dependent changes inaccessible for QTL analysis. Moreover, non-stable changes may also be spotted due to the usage of no replicates for ChIP-Seq studies, which may lead to spurious associations. In contrast, the model system we used is unique in that the confounding factors such as differences in LCL generation practice, sex, age, passage number and culture conditions can largely be excluded; therefore, it has the potential to investigate molecular phenotype differences independent from both direct and indirect genetic effects. Moreover, we found that there is a considerable difference between the H3K27ac signals of biological replicates, highlighting the importance of using biological replicates to exclude random fluctuations in association studies, which might otherwise lead to spurious associations.

The foundations of cellular heterogeneity in a single tissue, the extent of which has become recognised with the emergence of single-cell studies, are laid during cellular development^56^. It has been hypothesised that a higher level of heterogeneity in immune cells provide evolutionary advantage, contributing to robustness against unpredictable external perturbations. Functional genomic assays using a bulk of cells are generally not able to resolve cellular features at the single cell level. Instead, signals originating from individual cells add up to the total measured signal intensity at any detected entity (e.g. ChIP-Seq peaks), even in clonally-selected cell lines^1^. Moreover, the changing cellular environment in isogenic ES cells may trigger distinctive cellular mechanisms in cells with different network states, such as Nanog expression induction or premature death of Nanog-low cells in 2i-treated ESC lines, leading to a perception of an overall induction of Nanog^2^. It has been suggested that the diversity of LCLs rapidly decrease and may reach monoclonality within two months of culture^57^, and that a large proportion of publicly available LCLs may be mono- or pauciclonal^58,59^, although the level of clonality is generally not considered during the selection of LCLs to study. Two of the LCLs used in this study showed a ratio of kappa (κ) and lambda (λ) chains expected of polyclonal cell populations (0.4–0.6; sGT_2 and sGT_3). The other three cell lines are probably oligoclonal (sGT_1 and sGT_5) or monoclonal (sGT_4) (Fig. 2a,b, Table 1). Variable clonality across the tested cell lines, despite identical handling, might have emerged as a result of random subsampling of CD21 + B cells during the initiation of cultures, and subsequent variation in growth rates of their descendant lineages. Therefore, the features of one or a few parental B cells will predominantly be represented in bulk sequencing studies, leading to chromatin and gene expression differences. Transcriptional regulation pathways might also be selectively triggered by various secreted lymphokines.

Various confounding factors have been proposed that may significantly affect LCL drug response, including EBV copy number, baseline ATP levels and growth rate, with conflicting evidence on the heritability of and genetic predisposition to the above traits^29,60,61^. Not surprisingly, chemotherapeutic-induced cytotoxicity, given the mode of action of such agents, is especially dependent on cellular growth rate. At this point, we cannot exclude the possibility that 5-FU response differences between two of our cell lines are influenced by unmeasured potential confounders. An additional limitation of the study is our limited ability to extrapolate our findings to estimate the level of functional genomic variation due to non-genetic factors in large panels of LCLs due to the availability of only a handful of cell lines for such studies. Ultimately, our results expand our knowledge on the LCL model system in terms of feasibility and limitations, providing insights into the chromatin- and gene expression-level variability of isogenic LCL cells. Our study aligns with the recent investigations on pharmacogenomic implications of clonal variability of another model cell line, namely MCF-7 cells^1^. These studies raise the importance of the phenotypic variability of isogenic cells from a pharmacogenomic point of view. LCLs will probably remain a powerful tool in finding associations between genomic variants, molecular phenotypes, drug response and diseases, given the revealed limitations are carefully considered during experimental design. Taking into consideration the findings of our study, as well as other studies relevant to the topic, we can give recommendations regarding the selection criteria and experimental handling of LCLs for drug screenings. In general, potential confounding variables such as cell line preparation, culturing condition and passage number differences should be avoided. Also, it is recommended to use LCLs prepared from individuals with the same gender, with similar age, and belonging to the same population (e.g. CEPH/UTAH). It is also advised to use biological replicates for all performed assays in order to exclude random phenotypic fluctuations. Our study suggests that including baseline RNA levels in pharmacogenomics studies would lead to more robust findings, as has been proposed in an earlier study, calling this approach the „triangle study model”^43^. A model could also be implemented where gene expression-drug response associations are uncovered first, followed by validations in an independent cohort of cell lines harbouring genotypes potentially affecting the expression or post-transcriptional regulation of selected RNAs. Clonality may also be used as a selection criterion prior to association studies.

## Materials and Methods

### Cell culture

Human B-lymphoblastoid cell lines derived from five different tubes of anticoagulated peripheral blood, drawn from the same 26-year-old CEPH/UTAH male (GM22647, GM22648, GM22649, GM22650 and GM22651; denoted as sGT_1 to sGT_5 throughout the manuscript, respectively), were obtained from Coriell Cell Repositories. Cells were cultured in RPMI-1640 (Sigma-Aldrich, cat. R0883) supplemented with 15 v/v% heat-inactivated FCS (Thermo Fisher Scientific, cat. 10270–106), 2 mM L-glutamine (Sigma-Aldrich, cat. G7513) and 1 v/v% penicillin-streptomycin (Sigma-Aldrich, cat. P4333). To ensure a continuous source of cells for experiments, a three-tiered biobank was generated (see Supplementary Methods). Cell numbers were set to 8 * 10^5^ per ml culturing medium twelve hours prior to experiments. In all cases, all cell lines were handled in parallel using the exact same reagents and equipment, and experiments were initiated at the same time-point of the day.

### Basic cell line characterisation

Cell supernatants were tested for mycoplasma using the PCR Mycoplasma Test Kit I/C from PromoKine (cat. PK-CA91-1096). Genomic DNA was isolated using High Pure PCR Template Preparation Kit (Roche Life Science, cat. 11796828001), and five STR regions were amplified with fluorescently labeled primers (PowerPlex S5 System, Promega, cat. TMD021) (Table 1); the ABI PRISM 3100-Avant Genetic Analyzer and the GeneMapper ID software (v4.1) were used for detecting amplification products and analysis, respectively (Department of Laboratory Medicine, Faculty of Medicine, University of Debrecen).

### Flow cytometry

Cell suspensions were analysed by eight-colour labeling. Saturating concentrations of directly conjugated antibody combinations were added to cell suspensions (1 * 10^6^) and incubated for 15 min in the dark at RT. Samples were washed in PBS and fixed with 1% paraformaldehyde/PBS (PFA). For intracellular staining, the procedure described for Intrastain (Dako Glostrup, Denmark) was strictly followed. Surface staining was executed before permeabilisation and intracellular staining. One hundred thousand events were acquired with the help of FACS Canto II flow cytometer (Becton Dickinson, San Jose, CA). Data were analysed by FACS Diva (Becton Dickinson Biosciences, San Jose, CA) and Kaluza Software version 1.2 (Beckman Coulter, Brea, CA). For antibodies, clones and and vendor information please refer to Supplementary Methods.

For cell cycle analysis and DNA-index calculation, cells were washed twice with PBS at RT, fixed with 70 v/v% ethanol at 4 °C and centrifuged at RT. Cell pellets were resuspended with RNase (0.5 ml of 2 mg/ml RNase in PBS) and Propidium-iodide/Triton-X-100/EDTA (0.5 ml working solution of 100 μg/ml Propidium-iodide, 1% Triton-X-100, 12.7 μM EDTA in PBS). Samples were incubated for 30 min in the dark at RT. 20,000 events were acquired with the help of FACS Calibur flow cytometer (Becton Dickinson, San Jose, CA). Data were analyzed with ModFit LT for Mac 2.0 (Becton Dickinson, San Jose, CA).

### Chromatin immunoprecipitation and ChIP-Seq library preparation

All ChIP experiments were initiated from the same freezing batch of cells. Biological duplicates were prepared from different outgrowths (freezing vials) of cells on different days. Crosslinked chromatin was immunoprecipitated with anti-histone H3 (acetyl K27) antibody (Abcam, cat. ab4729) or isotype control antibody (Santa Cruz Biotechnology, cat. sc-2027X). Library preparation was performed by the Genomic Medicine and Bioinformatics Core Facility at the University of Debrecen, Debrecen, Hungary from 10 ng of ChIP material based on the “TruSeq ChIP Sample Preparation Guide 15023092 B” with minor modifications. Cluster generation, sequencing (50-bp, single-end) and demultiplexing (bcl2fastq Conversion Software) were performed either at the Genomic Medicine and Bioinformatic Core Facility at the University of Debrecen or at the EMBL Genomics Core Facility, Heidelberg, Germany. For detailed information about the in-house ChIP buffers and the experimental protocol, please refer to Supplementary Table 3 and Supplementary Methods, respectively.

### ChIP-Seq read alignment and data analysis

BWA 0.7.10 was used to align ChIP-Seq reads to the hg19 (GRCh37) genomic build. HOMER 4.9.1. was used to predict genomic regions with H3K27ac enrichment, and bedtools was used to remove sites overlapping ENCODE’s ‘blacklisted’ genomic regions. We used two-way ANOVA followed by Tukey’s post hoc test with functions aov() and TukeyHSD() from MASS package to identify significantly differentially enriched regions (P < 0.05, fold-change >2). For clustered enhancer prediction, all ChIP-Seq alignment (bam) files were merged using SAMtools (resulting in a total of ~200 million reads), a tag directory was created using HOMER’s makeTagDirectory program and clustered enhancers were predicted (HOMER’s findPeaks, options: -style super -L 0, -superSlope -1000, otherwise default parameters were used). We used the R package DiffBind (Bioconductor) to define a consensus set of predicted enhancers, calculate RPKM values, cluster the samples and create a correlation matrix. The correlation heatmap was created using plotly 3.0.0. (Python). We used the R package pheatmap to clusterize (k-means) differentially enriched regions based on fold-change to the first sample. The read distribution heatmap was generated by HOMER’s annotatePeaks (-hist function), centering to the nucleosome-free region (predicted from pooled bam files using HOMER’s getPeakTags with -nfr function) closest to the midpoint of each predicted region, and visualised using Java TreeView. The closest expressed genes were assigned to promoters, typical enhancers, and clustered enhancers using bedtools. We used the Integrative Genomics Viewer (IGV, Broad Institute) to visualise bedgraphs (created from merged bam files per sample), at selected genomic regions. We used the 3D Genome Browser from Yue Lab (http://promoter.bx.psu.edu/hi-c/index.html) to visualise HiC-based chromatin contacts in the GM12878 cell line with 40 Kb resolution and PhenoGram^62^ to visualise differentially enriched H3K27ac regions across chromosome 3 between sGT_1 and sGT_2.

### Total RNA isolation and mRNA-Seq library preparation

Total RNA was extracted from two million cells with TRIzolate reagent in biological duplicates (originating from two different growths of cells from the same tier of the biobank). For details, see Supplementary Methods. RNA concentration and sample purity were determined using a NanoDrop 1000 instrument (Thermo Fisher Scientific, Waltham, MA, USA). For the analysis of fragment distribution and calculation of RIN values (calculated being at least 9.4; mean: 9.8), RNA samples were loaded to Agilent RNA 6000 Nano microchips (Agilent, Santa Clara, CA, USA). Sequencing libraries were prepared following Illumina’s TruSeq RNA Sample Preparation v2 Guide with poly(A) selection using 1 μg total RNA as the starting material. Indexed libraries were pooled and subjected to single-end sequencing on a NextSeq500 sequencer (Illumina, San Diego, CA, USA) with 50-bp read length. Library preparation, cluster generation, sequencing and base calling were performed at the Genomic Medicine and Bioinformatic Core Facility at the University of Debrecen, Hungary. Demultiplexing was performed using the bcl2fastq Conversion Software (Illumina).

### RNA-Seq read alignment and data analysis

RNA-Seq reads were aligned to the hg19 (GRCh37) genomic build using TopHat v2.0.7 (–max-multihits option set to 1). Transcript abundances were calculated and batch effect was accounted for using edgeR and UCSC gene annotation track (hg19, downloaded from Illumina’s iGenomes database in 07/17/2015), and are expressed as RPKM values. Genes with CPM values (read counts per million mapped reads) below 5 across all samples were considered unexpressed and were discarded. We used edgeR to find genes that were differentially expressed between at least two cell lines (FDR = 0.05, FC > 2). The DAVID Bioinformatics Resources 6.8 tool was used for the functional annotation of differentially expressed genes (https://david.ncifcrf.gov/)^63,64^.

### RT-qPCR

Total RNA samples were treated with RQ1 DNase as per the manufacturer’s recommendations (Promega, cat. M6101), and were reversely transcribed using the SuperScript II system (Thermo Fisher Scientific, cat. 18064014). RT reactions were diluted and were subjected to qPCR using the LightCycler 480 SYBR Green I Master (Roche Applied Science, cat. 04887352001). QPCR measurements were carried out in triplicates. Expression levels were quantified using the ΔCp method and were normalized to *ACTB* expression. For additional details, see Supplementary Methods.

### 5-FU treatment

Cells in log-growth phase (2 * 10^5^) were treated in 96 U-well plates with two-fold serial dilutions of 5-FU (TEVA Pharmaceutical Industries, OGYI-T-4272/07) in indicator-free RPMI (Sigma-Aldrich, cat. R7509) supplemented with 15 v/v% heat-inactivated FCS, 2 mM L-glutamine and 1 v/v% penicillin-streptomycin. Cells were incubated at 37 °C (5% CO_2_) for 72 hours, resuspended with MTT stock solution (Sigma-Aldrich, cat. M5655), and incubated in the dark at 37 °C for 6 hours. Cell pellets were resuspended in 100 µl Lysis Solution and incubated for 1 hour. Absorbances at 595 nm were then measured. For additional details, see Supplementary Methods.

### Data visualisation

Data visualisations throughout the paper were performed using either Microsoft Excel (Microsoft Office Professional Plus 2013) or GraphPad Prism version 7.04 for Windows (GraphPad Software, La Jolla California, USA); www.graphpad.com.

## Supplementary information

Supplementary Information

Supplementary Information 1

Supplementary Information 2

## Data Availability

The ChIP-Seq and RNA-Seq data have been deposited in the GEO database under accession GSE121926.
