# Supplementary material for: Extensive epigenetic and transcriptomic variability between genetically identical human B-lymphoblastoid cells with implications in pharmacogenomics research
Source: Sci Rep. 2019 Mar 20;9:4889. doi: 10.1038/s41598-019-40897-9 (PMC6426863; doi:10.1038/s41598-019-40897-9)

Supplementary Information

Extensive epigenetic and transcriptomic variability between genetically identical human B-lymphoblastoid cells with implications in pharmacogenomics research

Lilla Ozgyin[1], Attila Horvath[1], Zsuzsanna Hevessy[2], and Balint L. Balint[1,*]

[1]Genomic Medicine and Bioinformatic Core Facility, Department of Biochemistry and Molecular Biology, Faculty of Medicine, University of Debrecen, H-4032 Debrecen, Hungary
[2]Department of Laboratory Medicine, Faculty of Medicine, University of Debrecen, H-4032 Debrecen, Hungary

Supplementary Methods

Biobank generation

A three-tiered biobank was generated for all LCLs used in this study, based on Coriell's cell culturing and Sigma-Aldrich's biobanking guidelines (http://www.sigmaaldrich.com/technical-documents/protocols/biology/good-cell-banking.html) in order to ensure a continuous source of seed stocks with an equally low number of cell passages and identical freeze-thaw history across cell lines. Cells were frozen in RPMI-1640 containing 20 v/v% heat-inactivated FCS and 6% DMSO (Hybri-Max, Sigma-Aldrich, cat. D2650) using an isopropanol bath at -60°C and transferred to the vapour phase of liquid nitrogen tanks. Prior to experimental use, cell batches from the third tier were reconstituted, passaged once at sub-confluence, and cell numbers were set to $8*10^5$ per ml culturing medium twelve hours prior to experiments.

Antibodies, clones and fluorochromes used for flow cytometry

	FITC	PE	PerCP-Cy5.5/PC5.5	PC7	APC	APC-AF750	PB	PO
**Tube 1.**	CD23 *(9P25)*	CD22 *(S-HCL-1)*	CD5 *(L17F12)*	CD19 *(J3-119)*	CD38 *(HB-7)*	CD81 *(JS-81)*	CD20 *(L27)*	CD45 *(HI30)*
**Tube 2.**	Kappa *(TB28-2)*	Lambda *(1-155-2)*	HLA-DR *(L243)*	CD19 *(J3-119)*	CD79b *(SN8)*	CD43 *(DFT1)*	FMC7 *(FMC7)*	CD45 *(HI30)*
**Tube 3.**	nTdT *(HT-6)*	cyIgM *(polyclonal)*	CD34 *(8G12)*	CD19 *(J3-119)*	CD10 *(HI10a)*	CD24 *(ML5)*	CD21 *(LT21)*	CD45 *(HI30)*

Abbreviations: nTdT: nuclear terminal deoxynucleotidyl transferase, cyIgM: cytoplasmic immunoglobulin M, FITC: fluorescein isothiocyanate, PE: phycoerythrin, PerCP-Cy5.5: peridinin chlorophyll protein 5.5, PC5.5: phycoerythrin cyanin 5.5 , PC7: phycoerythrin cyanin 7, APC: allophycocyanin, APC-AF750: conjugation allophycocyanin-alexa fluor 750, PB: pacific blue, PO: pacific orange

CD5, CD10, HLA-DR, CD20, CD22, CD24, CD34, CD38, CD79b, CD81, FMC7, HLA-DR, kappa and lambda markers were purchased from Becton Dickinson Biosciences (San Jose, CA); CD19, CD23, and CD43 markers were purchased from Beckman Coulter (Brea, CA); CD21 and CD45 markers were purchased from Exbio (Prague, Czech Republic); and nTdT and IgM were the products of Dako (Glostrup, Denmark).

Chromatin immunoprecipitation and ChIP-Seq library preparation

For the trio LCLs, 20 million cells were crosslinked at RT, first with 2 mM di(N-succinimidyl) glutarate (diluted from DSG with PBS, Sigma-Aldrich, cat. 80424-5MG-F) for 45 min and then with 1 % methanol-free formaldehyde (diluted from Pierce 16 v/w% Formaldehyde with PBS, Thermo Fisher Scientific, cat. 28908) for 10 min. For the sGT LCLs, 20 million cells were crosslinked at RT with 1 % methanol-free formaldehyde. Formaldehyde was quenched for 5 min at RT using 0.125 M glycine/PBS and cells were washed with ice-cold PBS. Nuclei were isolated by thoroughly resuspending cells in ChIP Lysis Buffer followed by high-speed centrifugation at 4°C (repeated 3 times) and sonicated using a probe-type sonicator (Bioruptor Plus, Diagenode) in ChIP Lysis Buffer. Nuclear debris was sedimented using high-speed centrifugation at 4°C, the top 90% of the supernatant was diluted ten-fold with ChIP Lysis Buffer, then diluted chromatin equivalent to 5 million cells were immunoprecipitated overnight at 4°C with 2.5 μg anti-histone H3 (acetyl K27) antibody (Abcam, cat. ab4729) or isotype control antibody (Santa Cruz Biotechnology, cat. sc-2027 X). Immunoprecipitation reactions were centrifuged and the top 90% of the supernatant (antibody-antigen complexes) was captured for 6 hours at 4°C using 47.5 μl Protein A-Protein G paramagnetic bead mix (1:1 ratio) (Thermo Fisher Scientific, cat. 10002D and 10004D) pre-blocked overnight with 0.5 w/v% BSA/PBS at 4°C. Capture beads were washed for 3 min on a rotating rack at 4°C once with IP Wash Buffer I, twice with IP Wash Buffer II, once with IP Wash Buffer III and

finally twice with IP Wash Buffer IV, collecting beads between washes using a magnetic rack (for the composition of in-house ChIP buffers please refer to Supplementary Table 3). Antibody-antigen complexes were eluted using 200 µl Bead Elution Buffer (30 min at RT, 1000 rpm), and crosslinks were reversed by adding 0.4 M NaCl and incubating overnight at 65°C. Eluates were then treated with 20 µg RNase A and 40 µg Proteinase K, respectively. ChIP fragments were isolated using Qiagen's MinElute PCR purification kit (cat. 28006) as per the manufacturer's recommendations. DNA concentrations were measured using the Qubit Fluorometer (Qubit dsDNA HS Assay Kit, Thermo Fisher Scientific, cat. Q32851), and enrichment at specific positive and negative control genomic sites were assayed by qPCR both before and after library preparation. The UPL Assay Design Center (Roche Applied Science, Germany) was used to design primer pairs, as well as appropriate Universal ProbeLibrary probes (Roche Applied Science, Germany) and reactions were run on a LightCycler 480 instrument.

RNA isolation

Cells were washed twice with PBS, carefully resuspended in 1 ml TRIzolate (UD-Genomed Medical Genomic Technologies Ltd., cat. URN0103) and vortexed for 5 min at RT. Phase separation was carried out using 200 µl chloroform (Sigma-Aldrich, cat. C2432) and high-speed centrifugation. RNA was precipitated from the aqueous phase using 750 µl isopropanol (Sigma-Aldrich, cat. I9516) and incubated for 10 min at RT. RNA pellets were washed twice with 1 ml chilled 75% ethanol (diluted with nuclease-free water from absolute ethanol, VWR International, cat. 20821.296), vacuum-desiccated and redissolved in nuclease-free water (AccuGENE, Lonza, cat. 51200) at 65°C for 10 min.

RT-qPCR

Residual DNA was removed from a 0.5 µg batch of total RNA using RQ1 DNase (2 U) as per the manufacturer's recommendations (Promega, cat. M6101), and was reversely transcribed using the SuperScript II system (Thermo Fisher Scientific, cat. 18064014). The reaction contained 1x FS buffer, 10 mM DTT, 0.5 mM dNTP mix, 0.8 U of SSII enzyme, 0.012 µg random hexamer primers and 0.125 µg DNase-treated RNA. The thermal profile of the RT reactions was as follows: 25°C for 10 min, 42°C for 50 min, followed by 70°C for 15 min. Control reactions lacking reverse transcriptase were also prepared for each sample. RT reactions were diluted five-fold with nuclease-free water and subjected to qPCR using the LightCycler 480 SYBR Green I Master (Roche Applied Science, cat. 04887352001) with 0.375 µM of each of the forward and reverse primers. The cycling parameters were 95°C for 10 min, followed by 50 cycles of 95°C for 10 s and 60°C for 30 s. The qPCR measurements were carried out in triplicates. Expression levels were quantified using the ΔCp method and were normalized to *ACTB* expression.

qPCR primers

DPYD and *ACTB* qPCR primers were designed using the Primer3Plus software and were analyzed using the OligoAnalyzer Tool (Integrated DNA Technologies).

**Gene Symbol**	**Forward (5' → 3')**	**Reverse (5' → 3')**
DPYD	CGTGTCAGAAGAGCTGTCCA	GGTCCCTCTTCAGTGGCATA
ACTB	CCCTGGCACCCAGCAC	GCCGATCCACACGGAGTAC

5-FU treatment

For 5-FU treatment, sGT_1 or sGT_2 cells in log-growth phase were washed with indicator-free RPMI medium (Sigma-Aldrich, cat. R7509) supplemented with 15 v/v% heat-inactivated FCS, 2 mM L-glutamine and 1 v/v% penicillin-streptomycin, and 2*105 cells in 50 µl phenol red-free RPMI were seeded in four wells of a 96 U-well plate (Sigma-Aldrich, M2186) per treatment type. The 5-FU stock solution was prepared from a 50 mg/ml original stock (TEVA Pharmaceutical Industries, OGYI-T-4272/07) using sterile ultrapure water, two-fold dilution series were prepared with indicator-free RPMI and added to the wells in 100 µl final volume. Cells were incubated undisturbed at 37°C (5% CO2) for 72 hours. The cells were carefully resuspended with 10 µl MTT stock solution (4.5 mg/ml thiazolyl blue tetrazolium bromide, dissolved in PBS) (Sigma-Aldrich, cat. M5655), the plate was sealed and wrapped into non-transparent foil and incubated at 37°C for 6 hours. Cell pellets were

resuspended in 100 µl Lysis Solution (20 w/v% SDS, 20 mM HCl in PBS), the plate was sealed again, wrapped into non-transparent foil and incubated for 1 hour. Absorbances at 595 nm were measured using a VICTOR3 Multilabel Plate Reader (PerkinElmer, MA, USA). Medium only wells were used as background.

Supplementary Table 1

Calculated PCR product size of each STR marker in sGT LCLs

Peak#	Marker	sGT_1	sGT_2	sGT_3	sGT_4	sGT_5
**1**	**Amelogenin (X,Y)**	98.33	98.31	98.33	98.22	97.95
**2**		104.36	104.34	104.31	104.3	104.0
**3**	**D18S51**	135.8	135.76	135.78	135.72	135.49
**4**		144.12	144.04	144.07	144.0	143.72
**5**	**D8S1179**	220.58	220.51	220.55	220.42	220.21
**6**		232.77	232.68	232.68	232.53	232.38
**7**	**TH01**	96.17	96.13	96.16	96.0	95.79
**8**		104.48	104.46	104.49	104.43	104.13
**9**	**FGA**	149.91	149.85	149.88	149.74	149.6
**10**		165.53	165.52	165.5	165.32	165.24

Supplementary Table 2

Selected ChIP-Seq statistics for each sGT LCL sample

Cell line	Replicate	Treatment	ChIPped histone mark	#Sequencing reads in fastq	Dup. reads (%)	Mapped reads (%)	IP efficiency[a]	#Peaks
**sGT_1 (GM22647)**	**1**	untreated	H3K27ac	20,243,528	0.80	97.9	22.0	28,882
	**2**	untreated	H3K27ac	27,016,541	1.74	98.1	21.3	29,253
**sGT_2 (GM22648)**	**1**	untreated	H3K27ac	13,914,920	0.78	97.8	22.4	29,108
	**2**	untreated	H3K27ac	28,146,360	1.22	98.2	20.6	30,511
**sGT_3 (GM22649)**	**1**	untreated	H3K27ac	14,997,414	0.78	97.8	22.2	28,233
	**2**	untreated	H3K27ac	34,480,788	1.29	98.3	21.0	30,539
**sGT_4 (GM22650)**	**1**	untreated	H3K27ac	16,598,015	1.00	97.6	23.2	29,149
	**2**	untreated	H3K27ac	25,277,925	1.51	98.2	20.3	29,722
**sGT_5 (GM22651)**	**1**	untreated	H3K27ac	13,682,731	0.98	97.7	21.9	27,625
	**2**	untreated	H3K27ac	23,488,876	1.35	98.4	21.2	28,805

[a] percent of mapped reads falling to regions of enrichment (peaks)

Supplementary Table 3

Composition of in-house ChIP-Seq buffers used in the study

Buffer name	Components
ChIP Lysis Buffer	1 v/v% Triton X-100 (Promega, cat. H5142)<br>0.1 w/v% SDS (VWR, 444464T)<br>150 mM NaCl<br>1 mM EDTA pH 8.0<br>20 mM Tris pH 8.0
IP Wash Buffer I	1 v/v% Triton X-100 (Promega, cat. H5142)<br>0.1 w/v% SDS (VWR, 444464T)<br>150 mM NaCl<br>1 mM EDTA pH 8.0<br>20 mM Tris pH 8.0<br>0.1 v/v% sodium-deoxycholate (VWR, cat. A1531.0100)
IP Wash Buffer II	1 v/v% Triton X-100 (Promega, cat. H5142)<br>0.1 w/v% SDS (VWR, 444464T)<br>500 mM NaCl<br>1 mM EDTA pH 8.0<br>20 mM Tris pH 8.0<br>0.1 v/v% sodium-deoxycholate (VWR, cat. A1531.0100)
IP Wash Buffer III	0.25 M LiCl<br>0.5 v/v% NP-40 (IGEPAL CA-630, Sigma-Aldrich, cat. I8896-50ML)<br>1 mM EDTA pH 8.0<br>20 mM Tris pH 8.0<br>0.5 v/v% sodium-deoxycholate (VWR, cat. A1531.0100)
IP Wash Buffer IV	10 mM EDTA pH 8.0<br>200 mM Tris pH 8.0
Bead Elution Buffer	100 mM $NaHCO_3$<br>1 w/v% SDS (VWR, 444464T)

Supplementary Fig. 1

H3K27ac variability within and between sGT LCLs. Scatter plots of RPKM values for each H3K27ac region belonging to the consensus peak set between **a** biological replicates of each sGT LCL and **b** between sGT LCLs. **c** R2 values calculated for each pair.

a

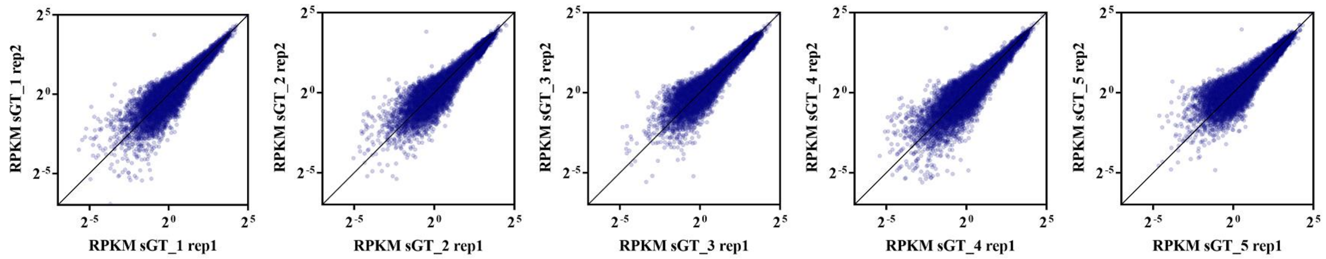

b

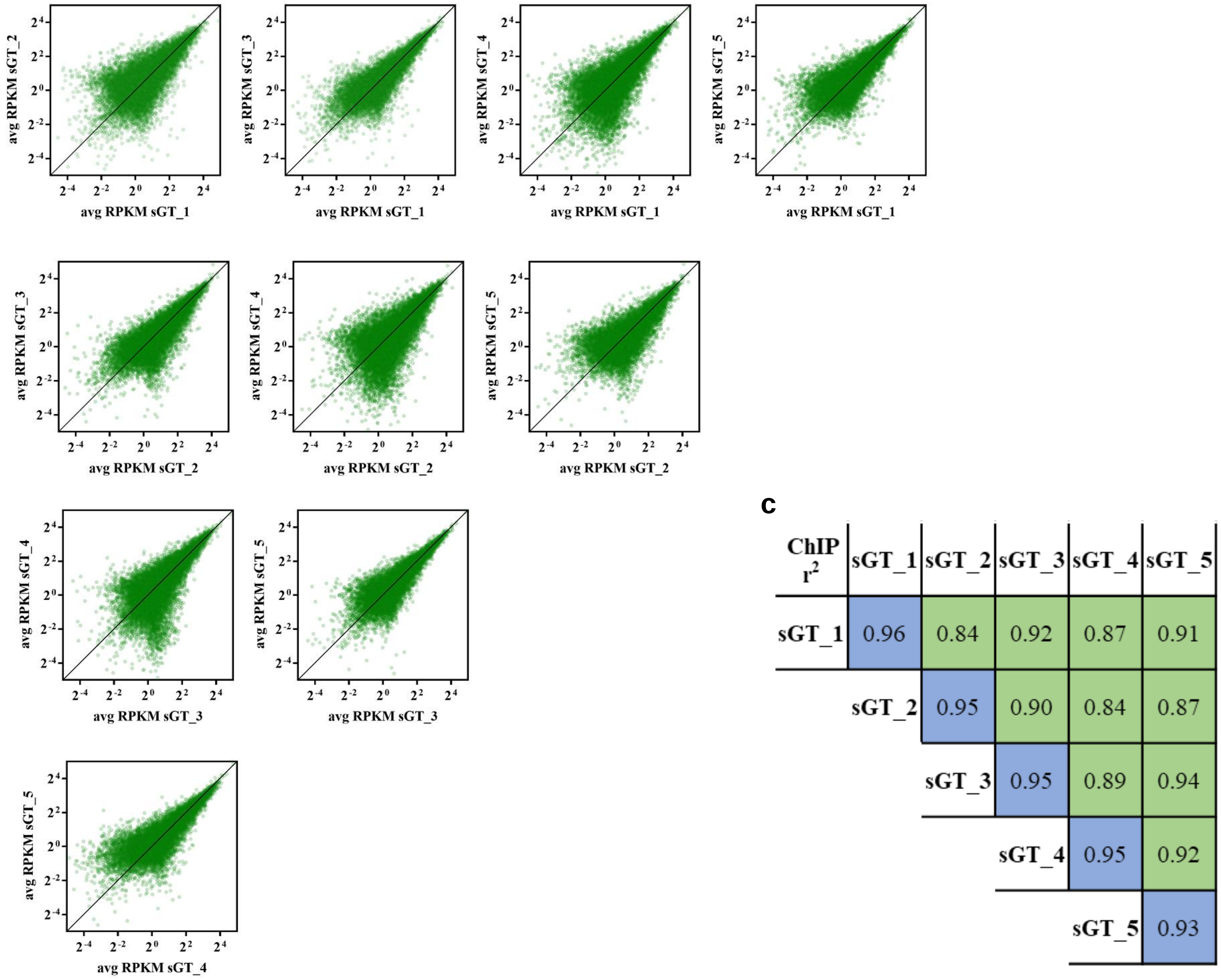

c

ChIP r^2	sGT_1	sGT_2	sGT_3	sGT_4	sGT_5
sGT_1	0.96	0.84	0.92	0.87	0.91
sGT_2		0.95	0.90	0.84	0.87
sGT_3			0.95	0.89	0.94
sGT_4				0.95	0.92
sGT_5					0.93

Supplementary Fig. 2

Gene expression variability within and between sGT LCLs. Scatter plots of RPKM values for each expressed gene **a** between biological replicates of each sGT LCL and **b** between sGT LCLs. **c** R2 values calculated for each pair.

a

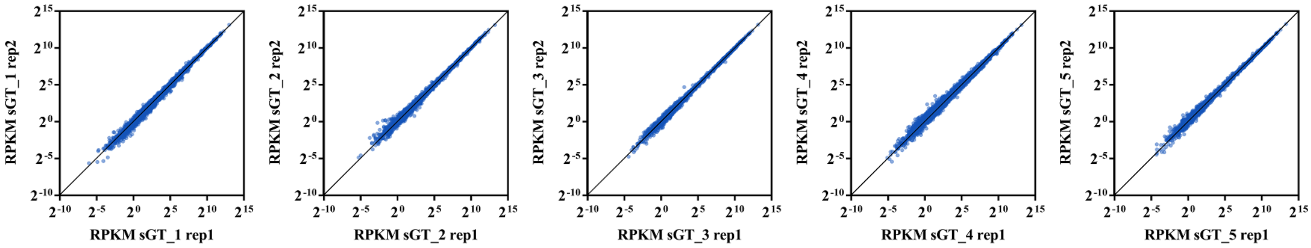

b

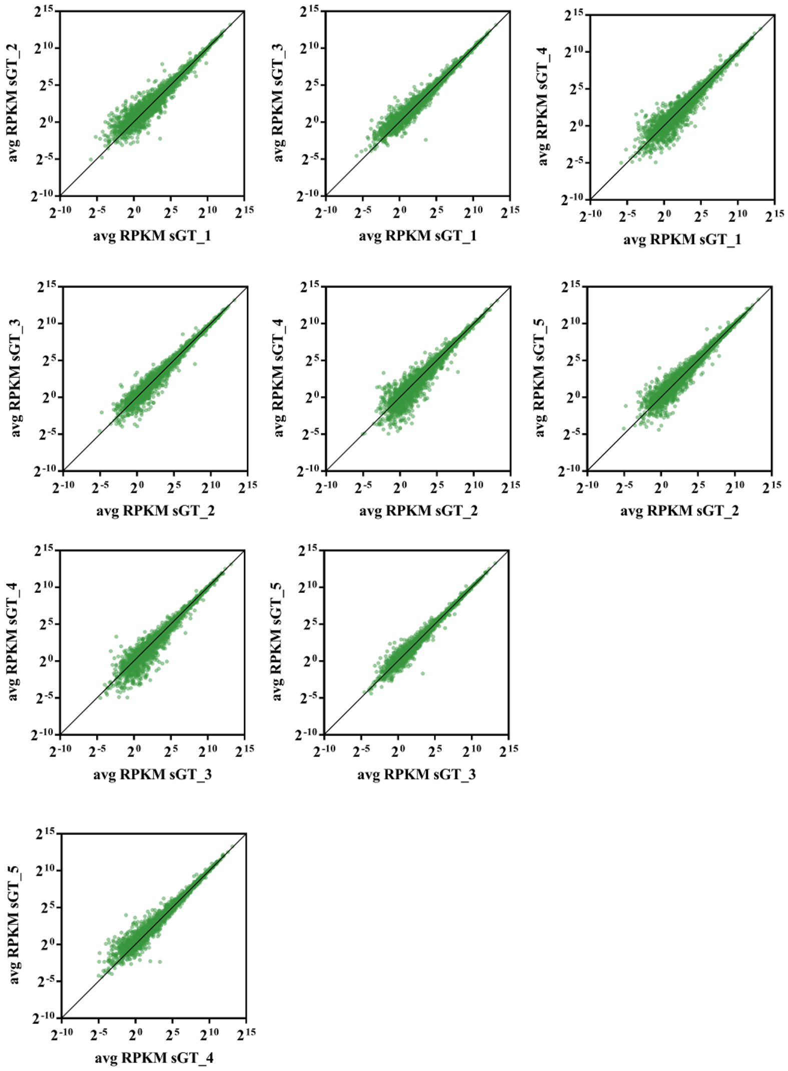

c

RNA r^2	sGT_1	sGT_2	sGT_3	sGT_4	sGT_5
sGT_1	0.996	0.991	0.997	0.991	0.993
sGT_2		0.995	0.993	0.990	0.988
sGT_3			0.999	0.993	0.996
sGT_4				0.996	0.993
sGT_5					0.999

Supplementary Fig. 3

GO annotation of genes belonging to the 1st, 2nd, 3rd, 4th and 5th quintiles of CV. –log10 P values of the most highly enriched **a** biological processes (BP), **b** cellular components (CC) and **c** molecular functions (MF) in each quintile.

a **Biological Process**

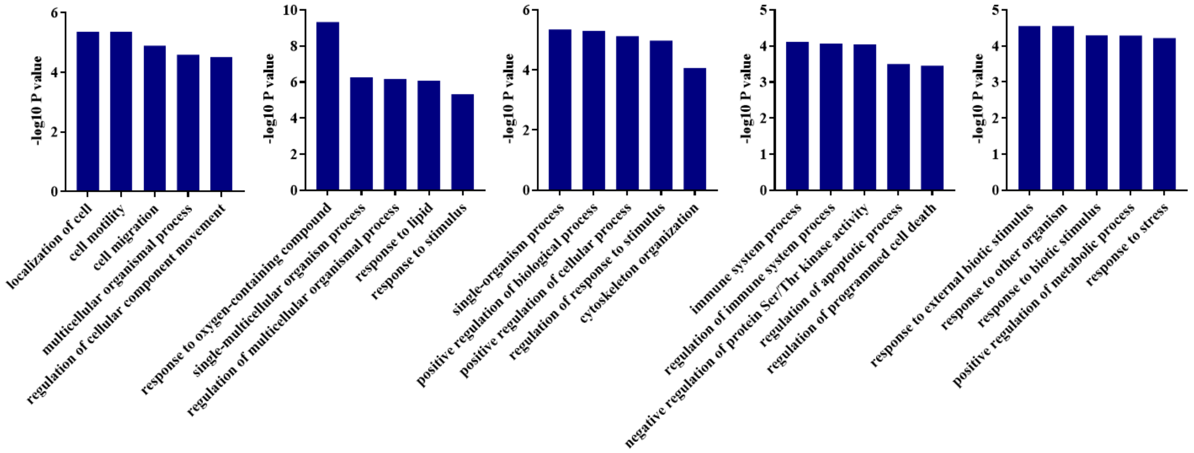

b **Cellular Component**

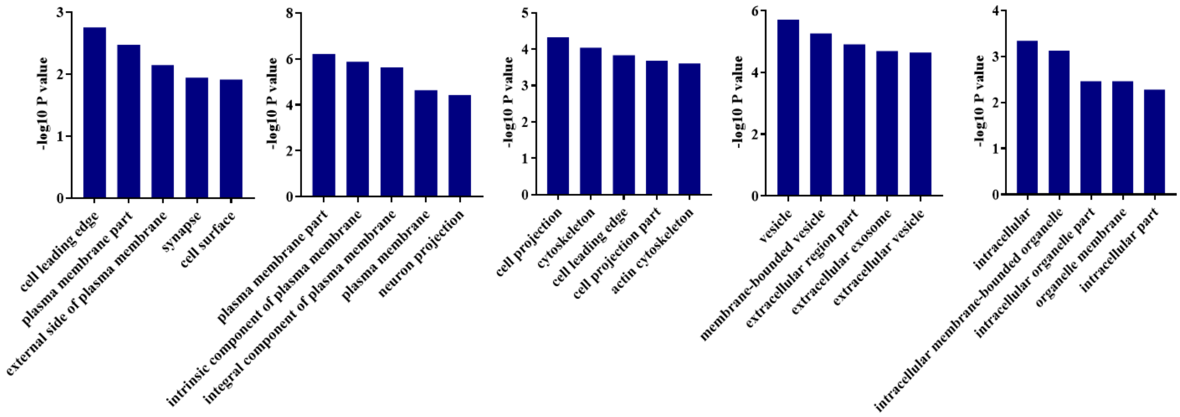

c **Molecular Function**

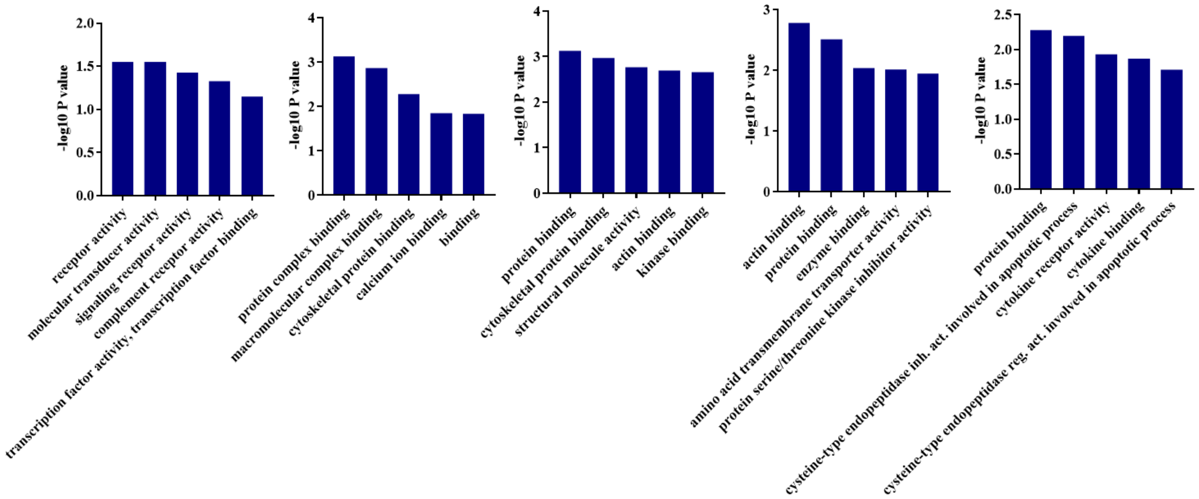

Supplement: Supplementary file 1 — Supplementary Information [file 41598_2019_40897_MOESM1_ESM.pdf]
