# Supplementary material for: Extensive epigenetic and transcriptomic variability between genetically identical human B-lymphoblastoid cells with implications in pharmacogenomics research
Source: Sci Rep. 2019 Mar 20;9:4889. doi: 10.1038/s41598-019-40897-9 (PMC6426863; doi:10.1038/s41598-019-40897-9)
Supplement: Supplementary file 2 — Supplementary Information 1 [file 41598_2019_40897_MOESM2_ESM.pdf]

Supplementary Information

Submitted through Nature Research's eProofing system

Manuscript title: 'Extensive epigenetic and transcriptomic variability between genetically identical human B-lymphoblastoid cells with implications in pharmacogenomics research'

Manuscript DOI: 10.1038/s41598-019-40897-9

The present file serves as a description of a replacement file for Figure 4, submitted as 'Supplementary_Information_2_Fig4' through the eProofing system.

In 'Supplementary_Information_2_Fig4' panels a, b, and c are unchanged. On panel d, the following changes have been made:

Original bottom part of panel d:

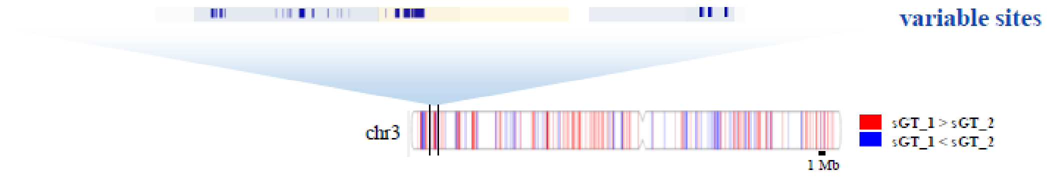

Modified bottom part of panel d:

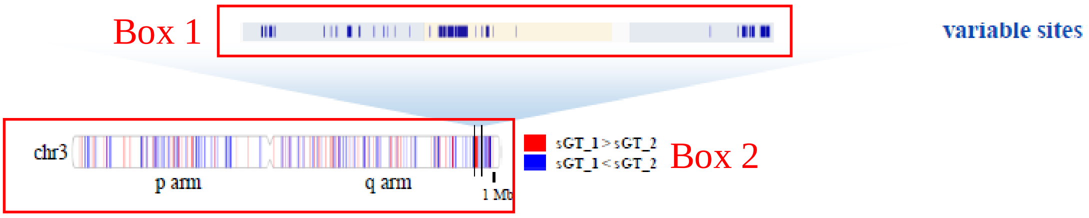

Box 1: The „variable sites" track has been replaced: the track now represents all variable sites across the cell lines detected with a P value treshold used throughout the manuscript for variable site detection ($P < 0.05$ and $FC > 2$ instead of $P < 0.01$ and $FC > 2$).

Box 2: The phenogram of chromosome 3 has been replaced: the region colors were wrongly assigned. Now, in agreement with the (unchanged) legend, red lines represent sites with significantly higher H3K27ac signal in sGT_1 than in sGT_2 ($P < 0.05$, $FC > 2$); blue lines represent sites with significantly higher H3K27ac signal in sGT_2 than in sGT_1 ($P < 0.05$, $FC > 2$). The phenogram has also been flipped vertically to represent the p arm -> q arm arrangement (arm names indicated).

Effect on the scientific content and conclusions of the manuscript:

The described changes **DO NOT** affect the conclusions of the manuscript, and **DO NOT** lead to changes to the manuscript text (neither in the main text, nor in the figure legend).
