# Supplementary figures and images for: Extensive epigenetic and transcriptomic variability between genetically identical human B-lymphoblastoid cells with implications in pharmacogenomics research
Source: Sci Rep. 2019 Mar 20;9:4889. doi: 10.1038/s41598-019-40897-9 (PMC6426863; doi:10.1038/s41598-019-40897-9)

**a**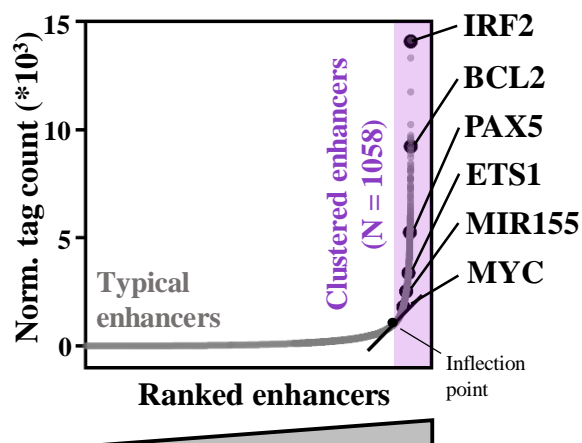**b**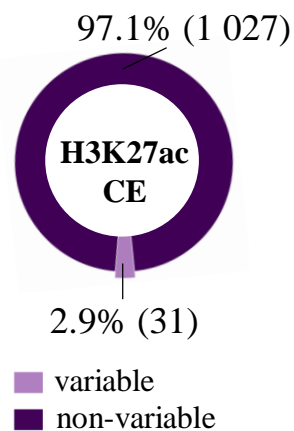**c**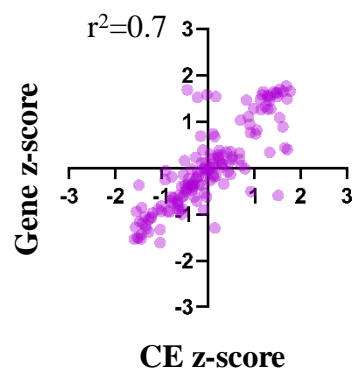**d**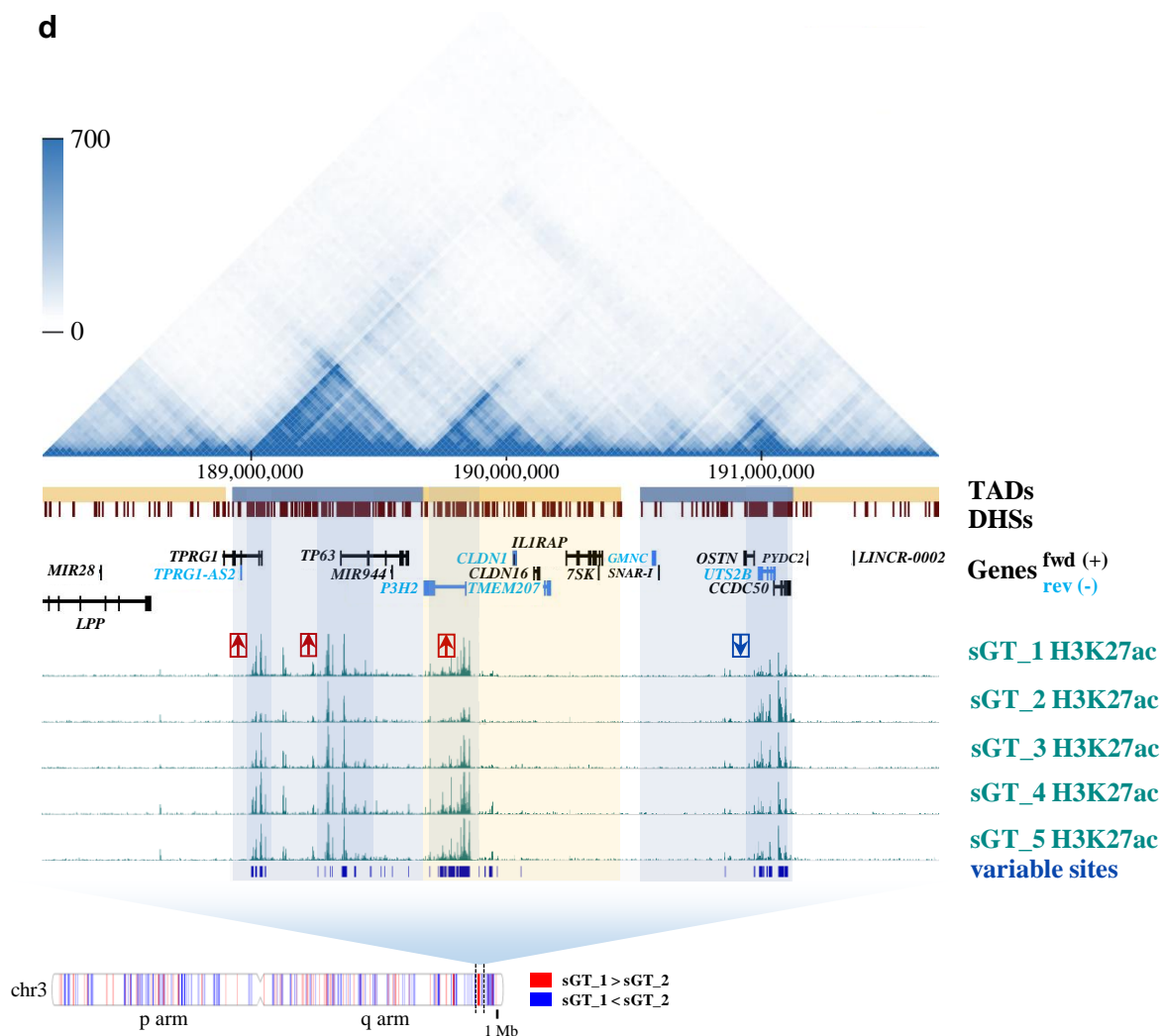

Supplement: Supplementary file 3 — Supplementary Information 2 [file 41598_2019_40897_MOESM3_ESM.pdf]
